# RNA interference against polo-like kinase-1 in advanced non-small cell lung cancers

**DOI:** 10.1186/2043-9113-1-6

**Published:** 2011-01-20

**Authors:** Eri Kawata, Eishi Ashihara, Taira Maekawa

**Affiliations:** 1 Department of Transfusion Medicine and Cell Therapy, Kyoto University Hospital, Kyoto, Japan; 2 Division of Internal Medicine, Kyoto Second Red Cross Hospital, Kyoto, Japan; 3 Department of Molecular Cell Physiology, Kyoto Prefectural University of Medicine, Kyoto, Japan

## Abstract

Worldwide, approximately one and a half million new cases of lung cancer are diagnosed each year, and about 85% of lung cancer are non-small cell lung cancer (NSCLC). As the molecular pathogenesis underlying NSCLC is understood, new molecular targeting agents can be developed. However, current therapies are not sufficient to cure or manage the patients with distant metastasis, and novel strategies are necessary to be developed to cure the patients with advanced NSCLC.

RNA interference (RNAi) is a phenomenon of sequence-specific gene silencing in mammalian cells and its discovery has lead to its wide application as a powerful tool in post-genomic research. Recently, short interfering RNA (siRNA), which induces RNAi, has been experimentally introduced as a cancer therapy and is expected to be developed as a nucleic acid-based medicine. Recently, several clinical trials of RNAi therapies against cancers are ongoing. In this article, we discuss the most recent findings concerning the administration of siRNA against polo-like kinase-1 (PLK-1) to liver metastatic NSCLC. PLK-1 regulates the mitotic process in mammalian cells. These promising results demonstrate that PLK-1 is a suitable target for advanced NSCLC therapy.

## Introduction

Worldwide, approximately one and a half million new cases of lung cancer are diagnosed each year [1]. About 85% of lung cancer are non-small cell lung cancer (NSCLC), including adenocarcinoma, squamous cell, and large cell carcinoma [2], and NSCLC is the leading cause of cancer-related deaths. Surgery is generally regarded as the best strategy for lung cancers. However, only 30% of patients are suitable for receiving potentially curative resection [3], and it is necessary for other patients to be treated with chemotherapy. As we gain a better understanding of the molecular pathogenesis underlying NSCLC, new molecular targeting agents can be developed. Tyrosine kinase inhibitors (TKIs) targeting the epidermal growth factor receptor (EGFR), such as gefitinib and erlotinib, have shown remarkable activity in the patients with NSCLC, and particularly these TKIs are more effective to NSCLC with *EGFR *mutations in 19 exon (in-frame deletions) and exon 21 (L858R point mutation), which are found to be more prevalent in Asian patients [4,5]. However, despite the development of new TKIs, new mutations in *EGFR *exon 20, developing resistance to *EGFR *TKIs, have emerged in the treated NSCLC [6,7], and current therapies are not sufficient to cure or manage the patients with distant metastasis [8,9]. Therefore, novel strategies are necessary to be developed so that the patients with NSCLC can be cured.

RNA interference (RNAi) is a process of sequence specific post-transcriptional gene silencing induced by double-strand RNA (dsRNA) and this phenomenon was discovered in *Caenorhabditis elegans *(*C. elegans*) [10]. RNAi has been shown to function in higher organisms including mammals, and methods that exploit RNAi mechanisms have been developing. RNAi has now been well-established as a method for experimental analyses of gene function *in vitro *as well as in high-throughput screening, and recently, RNAi has been experimentally introduced into cancer therapy. To apply the RNAi phenomenon to therapeutics, it is important to select suitable targets for the inhibition of cancer progression and also to develop effective drug delivery systems (DDSs). Recently a lot of useful non-viral DDSs for small interfering RNAs (siRNAs) have been developed [11-17]. Besides selecting suitable targets, an important consideration for siRNA-mediated treatment is to predict and avoid off-target effects, which are the silencing of an unintended target gene, and potential immunostimulatory responses. To avoid those effects, the most specific and effective siRNA sequence must be validated. Modification of two nucleosides of the sense strand also completely co-inhibited the immunological activities of the antisense strand, while the silencing activity of the siRNA was maintained [18].

Polo-like kinase-1 (PLK-1) belongs to the family of serine/threonine kinases and regulates cell division in the mitotic phase [19,20]. PLK-1 is overexpressed in many types of malignancies and its overexpression is associated with poor prognosis of cancer patients [21,22]. In this review, we discuss possible RNAi strategies against PLK-1 in advanced lung cancers.

### Mechanisms of RNAi

The precise mechanisms of RNAi are discussed in several reviews [23-25]. In the initiation phase of RNAi processes, following introduction of dsRNA into a target cell, dsRNA is processed into shorter lengths of 21-23 nucleotides (nts) dsRNAs, termed siRNAs, by the ribonuclease activity of a dsDNA-specific RNAse III family ribonuclase Dicer. Dicer consists of an N-terminal helicase domain, an RNA-binding Piwi/Argonaute/Zwille (PAZ) domain, two tandem RNAse III domains, and a dsRNA-binding domain [26,27]. Mammals and nematodes have only a single Dicer, which acts to produce both siRNAs and miRNAs [28-30], while other organisms have multiple Dicers which perform separate, specialized functions. *Drosophila *has two Dicers: *Drosophila *Dicer-1 is required for generating miRNAs, whereas *Drosophila *Dicer-2 produces siRNAs [25,31]. dsRNA precursors are sequentially processed by the two RNAse III domains of Dicer, and cleaved into smaller dsRNAs with 3' dinucleotide overhangs [26,32].

**Figure 1:**
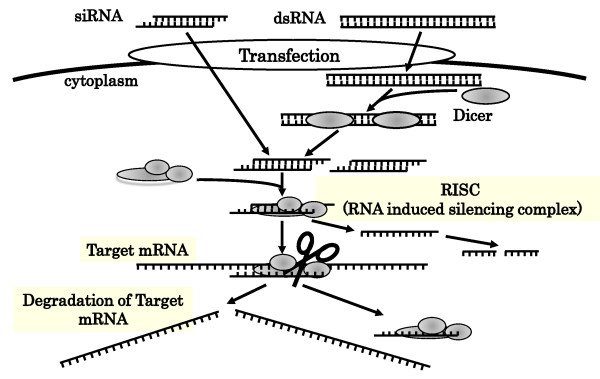
**Mechanisms of RNA interference**. After the introduction of dsRNA into a target cell, the dsRNA is processed into siRNA length of 21-23 nucletides by Dicer. siRNA then enters an RNA-induced silencing complex (RISC) assembly pathway. The dsRNA unwinds to form two single-strands of RNA. The passenger strand rapidly degrades and the guide strand binds and cleaves the target mRNA, resulting in mRNA degradation.

In the second effector phase, smaller dsRNAs enter into an RNA-induced silencing complex (RISC) assembly pathway [33]. RISC contains Argonaute (Ago) proteins, a family of proteins characterized by the presence of a PAZ domain and a PIWI domain [34]. The PAZ domain recognizes the 3' terminus of RNA, and the PIWI domain adopts an RNAse H-like structure that can catalyze the cleavage of the guide strand. Most species have multiple Ago proteins, but only Ago2 can cleave its RNA target in humans. The dsRNA is unwound by ATP-dependent RNA helicase activity to form two single-strands of RNA. The strand that directs silencing is called the guide strand, and the other is called the passenger strand. Ago2 protein selects the guide strand and cleaves its RNA target at the phosphodiester bond positioned between nucleotides 10 and 11 [32,35]. The resulting products are rapidly degraded because of the unprotected ends, and the passenger strand is also degraded [36,37]. The targeted RNA dissociates from the siRNA after the cleavage, and the RISC cleaves additional targets, resulting in decrease of expression of the target gene (Figure 1) [38].

### Polo-like kinase-1

To develop RNAi therapy against cancers, it is essential that suitable gene targets are selected. Such targets include antiapoptotic proteins, cell cycle regulators, transcription factors, signal transduction proteins, and factors associated with malignant biological behaviors of cancer cells. All of these genes are associated with the poor prognosis of cancer patients. PLKs belong to the family of serine/threonine kinases and are highly conserved among eukaryotes. PLK family has identified PLK-1, PLK-2 (SNK), PLK-3 (FNK), and PLK-4 (SAK) in mammalians so far and PLKs function as regulators of both cell cycle progression and cellular response to DNA damage [19,39-41]. PLK-1 has an N-terminal serine/threonine protein kinase domain and two polo box domains at the C-terminal region. Polo box domains regulate the kinase activity of PLK-1 [21,42]. PLK-1 regulates cell division at several points in the mitotic phase: mitotic entry through CDK1 activation, bipolar spindle formation, chromosome alignment, segregation of chromosomes, and cytokinesis [19,43]. *PLK-1 *gene expression is regulated during cell cycle progression, with a peak level occurring at M phase. Similar to its gene expression, PLK-1 protein expression and its activity are low in G0, G1, and S phases, and begin to increase in G2 phase with peak in M phase [44-47].

Whereas PLK-1 is scarcely detectable in most adult tissues [45,48,49], PLK-1 is overexpressed in cancerous tissues. Its expression levels were tightly correlated with histological grades of tumors, clinical stages, and prognosis of the patients. PLK-1 mRNA levels were elevated in NSCLC tissues and this transcript levels were correlated with the survivals of cancer patients [50]. Moreover, the immunohistoligical study showed that PLK-1 protein was overexpressed in NSCLC tissues in patients at progressed stages of cancer (postsurgical stage ≥II) and in patients with poorly differentiated NSCLCs [51]. Patients with urinary bladder cancers expressing high levels of PLK-1 have a poor prognosis compared with patients with its low expression. Moreover, the histologically high-grade, deeply invasive, lymphatic-invasive, and venous-invasive bladder cancers demonstrated significantly higher PLK-1 expression [52]. As PLK-1 is overexpressed in other various cancers [21], PLK-1 overexpression is a prognostic biomarker for cancer patients.

Inhibition of PLK-1 activity induces mitotic arrest and tumor cell apoptosis [53-55]. Depletion of *PLK-1 *mRNA also inhibits the functions of PLK-1 protein in DNA damages and spindle formation and causes the inhibition of the cell proliferation in a time- and a dose-dependent manner. PLK-1 siRNA treatment induces an arrest at the G2/M phase in the cell cycle with the increase of CDC2/Cyclin B1 [51,52,56,57]. PLK-1 siRNA-transfected cells had dumbbell-like and misaligned nuclei, indicating that PLK-1 depletion induced abnormalities of cell division during M phase, and these cells were shown to yield to caspase-dependent apoptosis [51,52,56]. As mentioned above, the kinases of PLK family cooperatively act in mitosis. Quantitative real-time RT-PCR data showed that PLK-2 and PLK-3 transcripts were increased after PLK-1 siRNA treatment [51]. Unlike PLK-1, PLK-2 and PLK-3 play inhibitory roles. PLK-2 is regulated by p53 and PLK-3 is activated by the DNA damage checkpoint [40]. These observations suggest that PLK-1 depletion induced mitotic catastrophe and activation of spindle checkpoint and DNA damage checkpoint, resulting in increased transcription of PLK-2 and PLK-3. Consequently, these PLK family kinases cooperatively prevented G2/M transition and induction of apoptosis. Importantly, depletion of PLK-1 does not affect the proliferation of normal cells although PLK-1 plays an important role in cell division [51,53,58]. This suggests that some other kinases compensate loss of PLK-1 function during mitosis in normal cells [51,58]. Collectively, PLK-1 could be an excellent target for cancer therapy.

### Atelocollagen

Although siRNA target molecules are overexpressed in cancer cells, most of them are essential to maintain homeostasis of physiological functions in humans. Therefore, siRNAs must be delivered selectively into cancer cells. Moreover, naked siRNAs are degraded by endogenous nucleases when administered *in vivo*, so that delivery methods that protect siRNAs from such degradation are essential. For these reasons, safer and more effective DDSs must be developed. DDSs are divided into two categories: viral vector based carriers, and non-viral based carriers. Viral vectors are highly efficient delivery systems and they are the most powerful tools for transfection so far. However, viral vectors have several critical problems in *in vivo *application. Especially, retroviral and lentiviral vectors have major concerns of insertional mutagenesis [59,60]. Consequently, non-viral DDSs have been strenuously developed [11-13].

Atelocollagen, one of powerful non-viral DDSs, is type I collagen obtained from calf dermis [61]. The molecular weight of atelocollagen is approximately 300,000 and the length is 300 nm. It forms a helix of 3 polypeptide chains. Amino acid sequences at the N- and C-termini of the collagen molecules are called telopeptide, and they have antigenecity of collagen molecules. As the telopeptide is removed from collagen molecules by pepsin treatment, atelocollagen shows low immunogenicity. Therefore, atelocollagen has been shown to be a suitable biomaterial with an excellent safety profile and it is used clinically for a wide range of purposes. Atelocollagen is positively charged, which enable binding to negatively charged nucleic acid molecules, and bind to cell membranes. Moreover, at low temperature atelocollagen exists in liquid form, which facilitates easy mixing with nucleic acid solutions. The size of the atelocollagen-nucleic acid complex can be varied by altering the ratio of siRNA to atelocollagen. Because atelocollagen naturally forms a fiber-like structure under physiological conditions, particles formed a high concentration of atelocollagen persist for an extended period of time at the site of introduction, which is advantageous to achieve a sustained release of the associated nucleic acid. Atelocollagen is eliminated through a process of degradation and absorption similar to the metabolism of endogenous collagen [61]. Alternatively, particles formed under conditions of low atelocollagen concentrations result in siRNA/atelocollagen complexes approximately 100-300 nm in size that are suitable for systemic delivery by intravenous administration. Atelocollagen complexes protect siRNA from degradation by nucleases and are transduced efficiently into cells, resulting in long-term gene silencing. For instance, Takeshita et al. demonstrated that the systemic siRNA delivery with atelocollagen existed intact for at least 3 days in tumor tissues using a mouse model [62].

### Preclinical application of RNAi therapy against PLK-1 in a murine advanced lung cancer model

**Figure 2:**
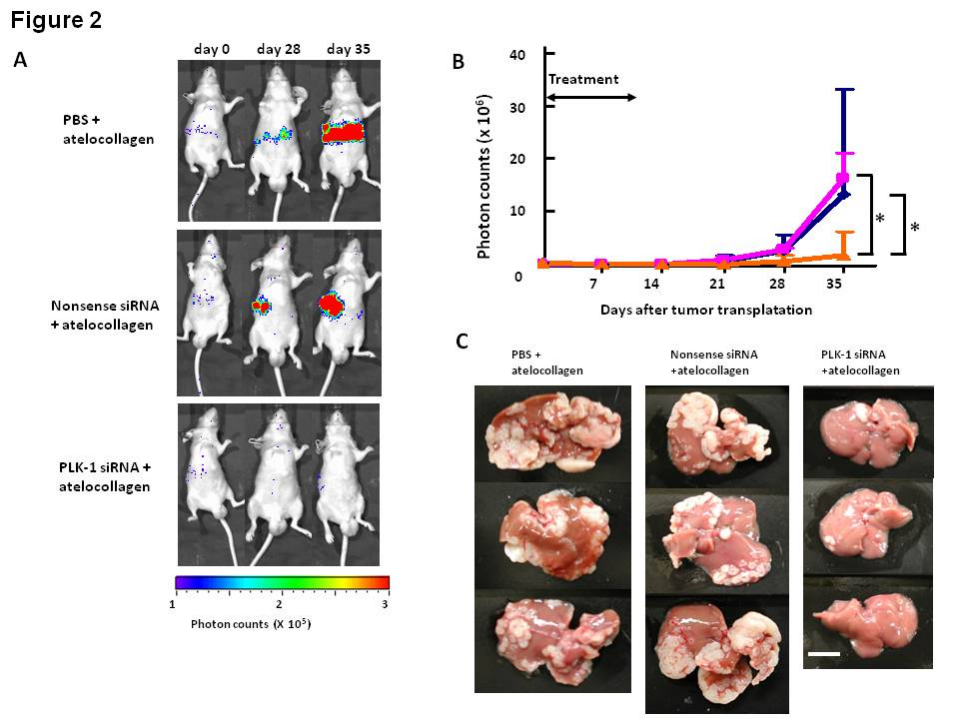
**Application of PLK-1 RNAi therapy against liver metastatic NSCLC (cited from **[51]**)**. A. PBS/atelocollagen complex, nonsense siRNA/atelocollagen complex, or PLK-1 siRNA/atelocollagen complex was administered by intravenous injection. Representative mice showing bioluminescence after siRNA treatment. The photon counts of each mouse are indicated by the pseudocolor scales. B. Growth curves of inoculated Luc-labeled A549 cells measured by the IVIS (pink square, nonsense siRNA/atelocollagen complex (25 μg siRNA)-treated mice; blue diamond, PBS/atelocollagen complex-treated mice; orange triangle, PLK-1 siRNA/atelocollagen complex (25 μg siRNA)-treated mice; n = 5 for each group. On day 35 after inoculation, the luminesecence in the PLK-1 siRNA/atelocollagen-treated mice was significantly suppressed compared with that in other groups. * *p *< 0.05. Mean ± SD. C. Macroscopic analysis of mice livers after day 70 of inoculation. White nodules are metastatic liver tumors. Treatment with PLK-1 siRNA (25 μg) remarkably inhibited the growth of liver metastases compared with PBS or nonsense siRNA treatments (25 μg).

Here we introduce an application of PLK-1 siRNA against an advanced lung cancer. As described above, PLK-1 is overexpressed in NSCLC tumors. Liver metastasis is one of the most important prognostic factors in lung cancer patients [8,9,63,64]. However, despite the development of new chemotherapeutic and molecular targeting agents, current therapies are not sufficient to inhibit liver metastasis. We investigated the effects of PLK-1 siRNA on the liver metastasis of lung cancers using atelocollagen as a DDS. We first established a mouse model of liver metastasis. Spleens were exposed to allow direct intrasplenic injections of Luciferase (Luc)-labeled A549 NSCLC cells. Ten minutes after injections of tumor cells, the spleens were removed. After Luc-labeled A549 cell engraftment was confirmed by using *In Vivo *Imaging System (IVIS) of bioluminescence imaging [65], PLK-1 siRNA/atelocollagen complex, nonsense siRNA/atelocollagen complex, or PBS/atelocollagen complex was administered by intravenous injection for 10 consecutive days following day 1 of transplantation. On day 35, mice treated with nonsense siRNA/atelocollagen complex or PBS/atelocollagen complex showed extensive metastases in the liver when compared to mice treated with PLK-1 siRNA/atelocollagen complex (Figure 2). Moreover, on day 70 after the inoculation of tumor cells, livers of mice treated with nonsense siRNA/atelocollagen or PBS/atelocollagen complex had numerous large tumor nodules, whereas the livers of mice treated with PLK-1 siRNA/atelocollagen complex showed a much lower number of smaller nodules. These findings indicate that PLK-1 siRNA/atelocollagen complex is an attractive therapeutic tool for further development as a treatment against liver metastasis of lung cancer [51]. Consequently, our preclinical applications suggest that PLK-1 siRNA is a promising tool for cancer therapy.

## Conclusion

Our preclinical studies demonstrated that RNAi therapy against PLK-1 using atelocollagen is effective against liver metastatic NSCLC cancers. Recently, several clinical trials for cancer therapy are ongoing (Additional file 1: Table S1, http://clinicaltrials.gov/ct2/home). Although RNAi shows excellent specificity in gene-silencing, several adverse effects including activation of immune reaction [66,67] and off-target effects (induction of unintended gene silencing) [68] are brought in *in vivo *application. Safer and more efficient DDSs for systemic delivery are warranted to be developed. Moreover, studies to establish the pharmacokinetics and pharmacodynamics of siRNAs on the administration are necessary steps in the potential approval of siRNA as a tool for cancer therapy. To maximize efficacy and to minimize adverse effects of RNAi, it should be determined whether siRNAs are best administered alone or in combination with chemotherapeutic agents [69,70], and whether it is better to administer a single specific siRNA or multiple specific siRNAs [57,71-73]. In conclusion, RNAi therapy represents a powerful strategy against advanced lung cancers and may offer a novel and attractive therapeutic option. The success of RNAi depends on the suitable selection of target genes and the development of DDSs. We anticipate that the continued development of effective DDSs and the accumulation of evidence further proving the success of siRNA treatment will advance RNAi as a promising strategy for lung cancer therapy.

## Lists of abbreviations

Ago: Argonaute; DDSs: drug delivery systems; dsRNA: double-strand RNA; EGFR: epidermal growth factor receptor; IVIS: *In Vivo *Imaging System; Luc: Luciferase; NSCLC: non-small cell lung cancer; nt: nucleotide; PAZ: Piwi/Argonaute/Zwille; PLK-1: Polo-like kinase-1; RISC: RNA-induced silencing complex; RNAi: RNA interference; siRNA: small interfering RNA; TKI: Tyrosine kinase inhibitor

## Competing interests

The authors declare that they have no competing interests.

## Authors' contributions

EK carried out our all experiments concerning this review and drafted the manuscript. EA designed our all experiments, carried out *in vivo *experiments, and wrote this review. TM supervised our research and wrote this review. All authors read and approved the final draft.

## Supplementary Material

Additional file 1 **Table S1 **Clinical trials of RNAi. Click here for file
