# Supplementary material for: RNA interference against polo-like kinase-1 in advanced non-small cell lung cancers
Source: J Clin Bioinforma. 2011 Jan 20;1:6. doi: 10.1186/2043-9113-1-6 (PMC3143898; doi:10.1186/2043-9113-1-6)
Supplement: Additional file 1 — Table S1 Clinical trials of RNAi. [file 2043-9113-1-6-S1.DOC]

Table 1 Clinical Trials of RNAi

| Company | siRNA | Target genes | Diseases | Administration | Phase | Year |
| --- | --- | --- | --- | --- | --- | --- |
| Opko Health | Bevasiranib | VEGF | AMD | Intravitreal | Phase III | 2005 |
|  |  |  | DME |  | Phase II | 2006 |
| Allergen | AGN-211745(sirna-027) | VEGFR1 | AMD | Intravitreal | Phase I/ II | 2006 |
|  |  |  | AMD |  | Phase I | 2006 |
| Quark | PF-4523655 ( RTP801i-14) | RTP801 | AMD | Intravitreal | Phase II | 2007 |
|  | QPI-1002 (AKIi-5) | p53 | Acute renal failure | Intravenous | Phase I | 2007 |
| Alnylam | ALN-VSP01 | RSV | RSV infection | Intranasal | Phase I | 2007 |
|  |  |  |  |  | Phase II | 2008 |
| TransDerm | TD101 | Keratin K6a | Pachyomychia Congenita | Plantar Calluses | Phase I | 2008 |
| **Calando** | **CALLA-01** | **RRM2** | **Solid tumors** | **Intravenous** | **Phase I** | **2008** |
| **Alnylam** | **ALN-VSP02** | **KSP + VEGF** | **Metastatic liver tumors** | **Intravenous** | **Phase I** | **2009** |
| **Silence Therapeutics** | **Atu027** | **PKN-3** | **Advanced solid tumors** | **Intravenous** | **Phase I** | **2009** |

Clinical trials of RNAi against cancers are described in boldface.

VEGF; vascular endothelial growth factor, RRM2; M2 subunit of ribonucleotide reductase, KSP; kinase spindle protein, PKN3; protein kinase N3, AMD; Age-related macular degeneration, DME; Diabetic macular edema, RSV; respiratory syncytial virus
